# Minibeam-pLATTICE: A novel proton LATTICE modality using minibeams

**Published:** 2025-02-27

**Authors:** Nimita Shinde, Weijie Zhang, Yuting Lin, Hao Gao

**Affiliations:** 1 Department of Radiation Oncology, University of Kansas Medical Center, Kansas City, USA

## Abstract

**Purpose:**

LATTICE, a form of spatially fractionated radiation therapy (SFRT) that delivers high-dose peaks and low-dose valleys within the target volume, has been clinically utilized for treating bulky tumors. However, its application to small-to-medium-sized target volumes remains challenging due to beam size limitations. To address this challenge, this work proposes a novel proton LATTICE (pLATTICE) modality using minibeams, namely minibeam-pLATTICE, that can extend the LATTICE approach for small-to-medium target volumes.

**Methods:**

Three minibeam-pLATTICE methods are introduced. (1) M0: a fixed minibeam aperture orientation (e.g., 0°) for all beam angles; (2) M1: alternated minibeam aperture orientations (e.g., between 0° and 90°), for consecutive beam angles; (3) M2: multiple minibeam aperture orientations (e.g., 0° and 90°) for each beam angle. The purpose of M1 or M2 is to correct anisotropic dose distribution at lattice peaks due to the planar spatial modulation of minibeams. For each minibeam-pLATTICE method, an optimization problem is formulated to optimize dose uniformity in target peaks and valleys, as well as dose-volume-histogram-based objectives. This optimization problem is solved using iterative convex relaxation and alternating direction method of multipliers (ADMM).

**Results:**

Three minibeam-pLATTICE methods are validated to demonstrate the feasibility of minibeam-pLATTICE for the head-and-neck (HN) patients. The advantages of this modality over conventional beam (CONV) pLATTICE are evaluated by comparing peak-to-valley dose ratio (PVDR) and dose delivered to organs at risk (OAR). All three minibeam-pLATTICE modalities achieved improved plan quality compared to CONV, with M2 yielding the best results. For example, in terms of PVDR, M2=5.89, compared to CONV=4.13, M0=4.87 and M1=4.7; in terms of max brainstem dose, M2=5.8 Gy, compared to CONV=16.57 Gy, M0=6.54 Gy and M1=7.04 Gy.

**Conclusions:**

A novel minibeam-pLATTICE modality is proposed that can generate lattice dose patterns for small-to-medium target volumes, which are not achievable with conventional pLATTICE due to beam size limitations. Peak dose anisotropy due to 1D planar minibeam apertures is corrected through inverse treatment planning with alternating or multiple minibeam apertures per beam angle.

## 1. Introduction

LATTICE radiation therapy [1], a form of spatially fractionated radiation therapy (SFRT) [2], divides the target volume into regions of high-dose (peak) and low-dose (valley) areas, delivering the dose according to the prescribed values for each. LATTICE can be viewed as a 3D extension of GRID [3]. LATTICE has been routinely used for treating bulky tumors with large target volumes [4]. However, applying LATTICE to small-to-medium target volumes remains challenging. This difficulty arises from the challenge of creating multiple well-separated peak regions, particularly when the target volume is small or located near critical organs at risk (OAR). A specific example is head-and-neck (HN) case, where the tumor is often close to the brainstem. In our experiments, it was observed that the conventional proton LATTICE radiation therapy delivered a maximum dose of 16.57 Gy to the brainstem.

To address this challenge, this work proposes the use of proton minibeam radiation therapy (pMBRT) [5, 6] for LATTICE radiation therapy. pMBRT utilizes multi-slit collimators (MSC) to generate sub-millimeter proton beams spaced a few millimeters apart, which makes it possible to create multiple well-separated peak regions within small-to-medium target volumes. While recent advancements have been made in conventional beam based proton LATTICE (pLATTICE) [7–10], minibeam based pLATTICE has not yet been explored.

This study introduces a novel minibeam-pLATTICE modality designed to deliver LATTICE radiation therapy to small-to-medium target volumes. Multiple models will be developed for to deliver minibeam-pLATTICE. A key question addressed here is the dose anisotropy in peak regions caused by the 1D planar dose modulation from the MSC. Specifically, inverse treatment planning methods incorporating alternating or multiple minibeam apertures per beam angle will be developed to correct for peak dose anisotropy introduced by planar minibeam apertures. As will be demonstrated in Section 3, while conventional proton pLATTICE delivered a maximum dose of 16.57 Gy to the brainstem in the HN case, the minibeam-pLATTICE model reduced the maximum dose to 5.8 Gy.

## 2. Methods and Materials

### 2.1. Optimization formulation for minibeam-pLATTICE

The inverse treatment planning problem for minibeam-pLATTICE can be formulated as

(1) $$\begin{array}{c} \underset{x}{\min}f(d)\\ \text{s.t.}\;x\in \{0\}\cup [G,+\infty \},\\ \text{d}=\text{A}x. \end{array}$$

In Eq. (1), the decision variable x represents the spot intensity vector, A represents the dose influence matrix, and d is the dose distribution. The first constraint in Eq. (1) is a minimum-monitor-unit (MMU) constraint [11, 12] that ensures plan deliverability by constraining the smallest non-zero value of x to be G. The second constraint in Eq. (1) defines the dose distribution.

The objective function, f(d), in Eq. (1) defines the least square error between the actual doses and the dose constraints and is mathematically defined as

(2) $$\begin{array}{c} f(d)=\frac{w_{p}}{n_{peak}}{\left\|d_{peak}-b_{peak}\right\|}_{2}^{2}+\frac{w_{v}}{n_{valley}}{\left\|d_{valley}-b_{valley}\right\|}_{2}^{2}+\frac{w_{1}}{n}{\left\|d_{{\text{Ω}}_{1}}-b_{1}\right\|}_{2}^{2}\\ +\sum_{\text{i}=1}^{{\text{N}}_{2}}\frac{{\text{w}}_{2}}{n_{i}}{\left\|{\text{d}}_{{\text{Ω}}_{2\text{i}}}-{\text{b}}_{2\text{i}}\right\|}_{2}^{2}+\sum_{\text{i}=1}^{{\text{N}}_{3}}\frac{w_{3}}{n_{i}}{\left\|{\text{d}}_{{\text{Ω}}_{3\text{i}}}-{\text{b}}_{3\text{i}}\right\|}_{2}^{2}+\sum_{\text{i}=1}^{{\text{N}}_{4}}\frac{w_{4}}{n_{i}}{\left\|{\text{d}}_{{\text{Ω}}_{4\text{i}}}-{\text{b}}_{4\text{i}}\right\|}_{2}^{2}. \end{array}$$

We now describe each term in f(d) briefly.

- In the first term of $f(d),b_{peak}$ and $d_{peak}$ are the prescribed dose and actual dose delivered to peak region in the target volume respectively. Thus, the first term defines the least square error between the prescribed and actual peak dose. Similarly, the second term of f(d) is the least square error between the actual valley dose $\left(d_{valley}\right)$ and prescribed valley dose $\left(b_{valley}\right)$. Note that, $n_{peak}$ and $n_{valley}$ are the number of voxels in the peak and valley regions of the target volume respectively.
- The third term in f(d) defines the error for dose volume histogram (DVH)-min constraint [13, 14] for the target. The DVH-min constraint ensures that at least p% of the total voxels in the target volume receive a dose larger than the minimum dose $b_{1}$. To define the DVH-min constraint, first define the active index set ${\text{Ω}}_{1}$ as ${\text{Ω}}_{1}=\{j|j\leq p\times n\}$ if $d_{p\times n}^{\prime }\leq b_{1}$, where $d^{\prime }$ is the dose distribution d sorted in descending order and n is the number of voxels in the target. The set ${\text{Ω}}_{1}$ consists of indices of target volume which violate the DVH-min constraint. The term, ${\left\|d_{{\text{Ω}}_{1}}-b_{1}\right\|}_{2}^{2}$, then defines the least square error between the actual dose and minimum dose $b_{1}$, for the indices in the active index set ${\text{Ω}}_{1}$.
- The fourth term describes $N_{2}$ DVH-max constraints [13, 14] for OAR. For any OAR i, the DVH-max constraint ensures that at most p% of the total voxels in OAR i receive a dose larger than $b_{2i}$. The active index set ${\text{Ω}}_{2i}$ is defined such that it contains indices of voxels in OAR i that violate the DVH-max constraint. Mathematically, ${\text{Ω}}_{2i}=\left\{j|j\geq p\times n_{i}\right\}$ if $d_{p\times n_{i}}^{\prime }\geq b_{2i}$, where $d^{\prime }$ is the dose distribution d sorted in descending order and $n_{i}$ is the number of voxels in OAR i. Thus, the fourth term in f(d) defines the error between actual dose and $b_{2i}$, for the voxels of OAR i that violate the DVH-max constraint.
- The fifth term in f(d) defines the least square error for OAR voxels that violates the D-max (dose-max) constraint. For any OAR i, the D-max constraint ensures that all the voxels in OAR i receive dose at most $b_{3i}$. If there exist voxels in OAR i that violate the constraint, then the set ${\text{Ω}}_{3i}=\{j\in \left[n_{i}\right]|d_{j}\geq b_{3i}\}$ is non-empty. Thus, the fifth term defines the error for the voxels in OAR i that violate the D-max constraint.
- The last term in f(d) defines the least square error for OAR that violate the D-mean (dose-mean) constraint. For any OAR i, the D-mean constraint ensures that the mean dose delivered to all voxels in OAR i is less than or equal to $b_{4i}$. If this constraint is satisfied, the active index set ${\text{Ω}}_{4i}$ is empty. However, if the constraint is violated, then ${\text{Ω}}_{4i}=\left[n_{i}\right]$, i.e., the active index set consists of all voxels in OAR i.

### 2.2. Optimization methods for minibeam-pLATTICE

This work introduces three minibeam-pLATTICE models (M0, M1 and M2), distinguished by the orientation of multi-slit collimators (MSC) relative to proton minibeams at different beam angles. Due to the highly directional nature of minibeams, the delivered dose exhibits anisotropic distribution within the target. To address this issue, this work explores using collimators with varying orientations to modulate the dose distribution, achieving a more uniform 3D peak dose pattern with spherical-shaped peak regions.

- **M0 (Baseline Model):** Utilizes a fixed horizontal collimator orientation (0° relative to the beam source) for all beam angles.
- **M1 (Alternating Orientation Model):** Alternates between two orthogonal collimator orientations at different beam angles. In this study, two orthogonal orientations of 0° and 90° with respect to the beam sources are used.
- **M2 (Multi-Collimator Model):** Employs multiple collimators at each beam angle. In this study, both 0° and 90° orientations are used simultaneously at each beam angle. This results in two distinct minibeam apertures per beam angle, helping to counteract anisotropic dose distribution.

### 2.3 Optimization algorithms for minibeam-pLATTICE

To solve Eq. (1), auxiliary variable for the MMU constraint is introduced, and Eq. (1) is re-written as

(2) $$\begin{array}{c} \underset{x}{\min}f(d)\\ \text{s.t.}\;z\in \{0\}\cup [G,+\infty \},\\ z=x\\ \text{d}=\text{Ax} \end{array}$$

Eq. (2) can be solved via iterative convex relaxation (ICR) method [15, 16] and alternating direction method of multipliers (ADMM) [17, 18]. This technique has been used to solve various inverse optimization problems in radiation therapy [19–28]. The iterative method described here involves updating the active index sets for the terms in f(d), followed by updating each decision variable in Eq. (2) sequentially while keeping other variables fixed. To use the ADMM method, define the augmented Lagrangian for Eq. (2) as

(3) $$\begin{array}{c} \underset{x,z}{\min}f(\text{Ax})+\frac{{\text{μ}}_{1}}{2}{\left\|z-x+\lambda_{1}\right\|}_{2}^{2}\\ \text{s.t.}\;z\in \{0\}\cup [G,+\infty \}. \end{array}$$

Algorithm 1 is then used to solve the augmented Lagrangian formulation.

**Algorithm 1:** Optimization method for solving Eq. (3)

1.	**Input:** Choose parameters ${\text{μ}}_{1}$, $w_{p}$, $w_{v}$, $w_{1}$, $w_{2}$, $w_{3}$, $w_{4}$
2.	Initialization: Randomly initialize x. Choose number of iterations T.
3.	Set $\lambda_{1}=z=x$.
4.	For t=1,…,T
	a. Find active index sets ${\text{Ω}}_{1}$, ${\text{Ω}}_{2i}$, ${\text{Ω}}_{3i}$, ${\text{Ω}}_{4i}$ as described in Section 2.1.
	b. Update primal variables x, z one at a time by fixing other variables and solving the resulting minimization problem.
	c. Update dual variable as $\lambda_{1}=\lambda_{1}+z-x$.
5.	**Output:** x

In Step 4b of Algorithm 1, x is updated by fixing the variable z. The resulting minimization problem is unconstrained in x. Thus, the optimal value of the decision variable x is obtained by taking first-order derivative of the objective function and solving the linear system of equations. Similarly, to update z in Step 4b, x is fixed. The minimization problem in z has the following closed form solution

$$z=\{\begin{array}{cc} max\left(G,x-\lambda_{1}\right), & \text{if}\;x-\lambda_{1}\geq G/2\\ 0, & otherwise. \end{array}$$

### 2.4 Materials

The performance of the conventional pLATTICE (CONV) model is compared with the three proposed minibeam-pLATTICE models, namely, M0, M1 and M2, for the clinical head-and-neck (HN) patients. Two HN cases are presented here. In the first case (HN01), the prescribed valley and peak doses are 2 Gy and 10 Gy, respectively. For the second case (HN02), these doses are 2.12 Gy and 10.6 Gy. The beam angles used are (0°, 45°, 90°, 135°, 180°, 225°, 270°, 315°) and (45°, 135°, 225°, 315°) for HN01 and HN02 cases respectively. For minibeam-pLATTICE models, 13 spherical peak regions (1.5 mm diameter) in HN01 and 14 in HN02 are defined. In contrast, the conventional pLATTICE model includes 2 spherical peaks (10 mm diameter) in both cases. Each minibeam-pLATTICE model utilizes a multi-slit collimator with a 7 mm center-to-center slit distance and 0.4 mm slit width. The collimator orientations for M0, M1, and M2 follow the configurations described in Section 2.2. Dose influence matrices for each beam angle and source are generated using MatRad [29], with a spot width of 0.4 mm on a 1×1×1 mm^3^ dose grid.

All plans are normalized to ensure that 95% of the valley region receives at least 100% of the prescribed valley dose. Plan quality is evaluated based on mean peak and valley doses, mean doses delivered to OAR, the normalized maximum peak dose, and peak-to-valley dose ratio (PVDR). The normalized maximum peak dose is calculated as ${\text{D}}_{\text{max}}=(\text{D}/{\text{D}}_{\text{pp}})\times 100\%$, D is the maximum dose delivered to peak region, and ${\text{D}}_{\text{pp}}$ is the prescribed peak dose, and PVDR is computed as: $\text{PVDR}={\text{D}}_{10}/{\text{D}}_{80}$ [30, 31], where ${\text{D}}_{10}$ and ${\text{D}}_{80}$ are the doses delivered to at least 10% and 80% of the entire target volume respectively.

## 3. Results

### 3.1 Conventional pLATTICE vs. minibeam-pLATTICE

From Tables 1 and 2, it is evident that for both HN cases, the mean peak and valley doses are closer to the prescribed doses in the minibeam-pLATTICE models (M0, M1, M2) compared to the conventional pLATTICE model (CONV). For HN01, the PVDR is improved for all three minibeam-pLATTICE models: M0 = 4.87, M1 = 4.7, and M2 = 5.89, while CONV has a PVDR of 4.13. Additionally, a significant reduction in the maximum dose delivered to the brainstem is observed for the minibeam-pLATTICE models (CONV = 16.75 Gy, M0 = 6.54 Gy, M1 = 7.04 Gy, M2 = 5.8 Gy) in HN01.

**Figure 2.**
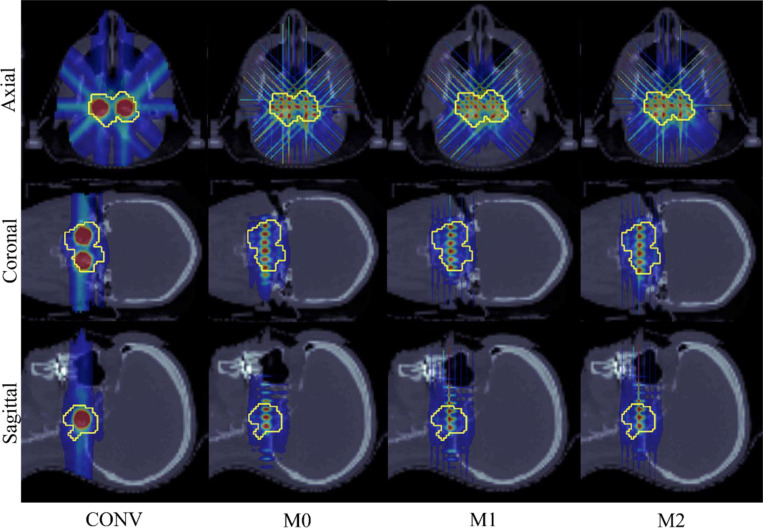
HN01. Dose plots: conventional pLATTICE (CONV) and minibeam-pLATTICE (M0, M1, M2).

**Figure 3.**
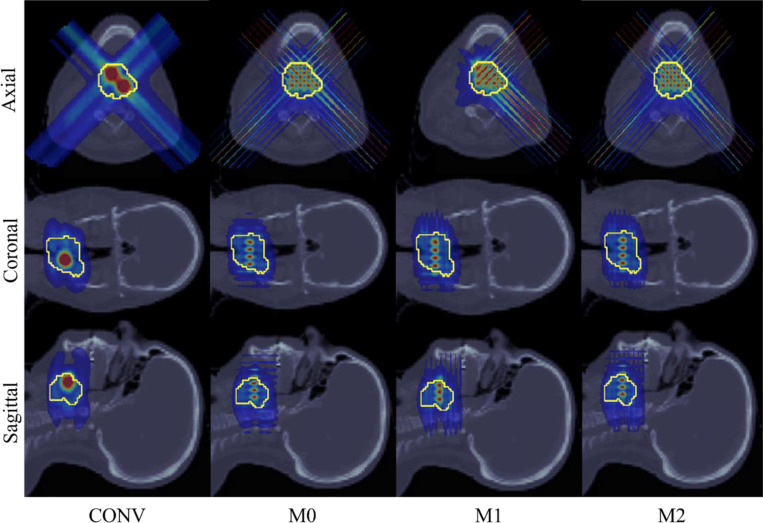
HN02. Dose plots: conventional pLATTICE (CONV) and minibeam-pLATTICE (M0, M1, M2).

For other OAR in HN01 and HN02, the maximum doses delivered to the OAR show a slight increase, though the mean doses remain similar across all methods. Furthermore, for HN01, Figure 2 demonstrates distinct spherical lattice peaks generated by the M2 model, with sufficient separation from the OAR. In contrast, for the CONV model, the peaks are closer to the OAR, resulting in higher doses to the brainstem. Similarly, Figure 3 shows that the M0 and M2 models in HN02 generate distinct peaks that are well separated from the OAR, while the peaks in the CONV model are closer to the tumor boundary. These observations highlight the advantage of minibeam-pLATTICE over conventional pLATTICE.

### 3.2 Comparison of different minibeam-pLATTICE methods

Within the minibeam-pLATTICE modality, three models are proposed as described in Section 2.2. From Tables 1 and 2, it is clear that the M2 model achieves the highest PVDR for both HN cases. For example, in HN02, the PVDR values are: M0 = 2.05, M1 = 2.31, and M2 = 2.55. A reduction in the maximum dose to the brain and brainstem is also observed for M2 in the HN01 case.

The performance of all three models in terms of mean doses delivered to the body and other OAR is similar. However, as seen in Figures 2 and 3, the M2 model generates distinct spherical peaks for HN01, while both M0 and M2 produce distinct peaks in HN02. These results lead to the conclusion that the M2 model, which employs multiple collimator orientations per beam angle, delivers the best plan quality.

## 4. Discussion

This study introduces a novel approach that integrates proton minibeam radiation therapy (pMBRT) with LATTICE radiation therapy (LRT) for treating small-to-medium-sized tumors. However, other aspects of proton minibeam therapy and LATTICE optimization remain unexplored.

Maintaining a high peak-to-valley dose ratio (PVDR) is crucial in LRT. Recent studies [31, 32, 33] propose a joint treatment planning approach that optimizes dose conformity and dose-volume histogram (DVH)-based constraints while maximizing PVDR. Their findings demonstrate that this joint optimization improves target dose uniformity while achieving high PVDR at organs at risk (OAR). Additionally, [31] introduces a multi-collimator pMBRT approach that employs a set of multi-slit collimators (MSC) with varying center-to-center (ctc) distances at each beam angle, offering provable advantages over single-collimator methods. Integrating such PVDR optimization techniques with the minibeam-pLATTICE modality could further enhance PVDR in OAR while maintaining or improving target dose distribution.

Another critical challenge in pencil beam scanning proton therapy is spot placement, which is particularly important in LRT to ensure sharp dose gradients near peak region boundaries. Proper alignment with MSC is also essential. Recent works [34, 35] explore adaptive strategies to optimize dose conformality and delivery efficiency, while [36] introduces a hybrid approach combining large and small spot sizes to enhance dose falloff at peak boundaries. Implementing such spot placement and spot size optimization techniques could significantly improve dose conformality in minibeam-pLATTICE.

While this study validates the feasibility of minibeam-pLATTICE through computational modeling, experimental validation [37, 38] is necessary to facilitate clinical translation.

Overall, this work demonstrates the potential of applying LRT to small-to-medium target volumes using proton minibeams. However, further research is required to refine dose optimization strategies, improve delivery efficiency, and validate clinical applicability.

## 5. Conclusion

This study aimed to extend the LATTICE method to treat small-to-medium-sized target volumes. To achieve this, a novel proton LATTICE approach using minibeams, minibeam-pLATTICE, was introduced. Three models were developed, each incorporating different collimator orientations relative to the proton beam source. The approach successfully generated dose plans with distinct lattice peak and valley regions for clinical head-and-neck cases with small target volumes. To the best of our knowledge, this is the first study to apply the LATTICE method to small-to-medium-sized tumors using proton minibeams.

## Figures and Tables

**Figure 1:**
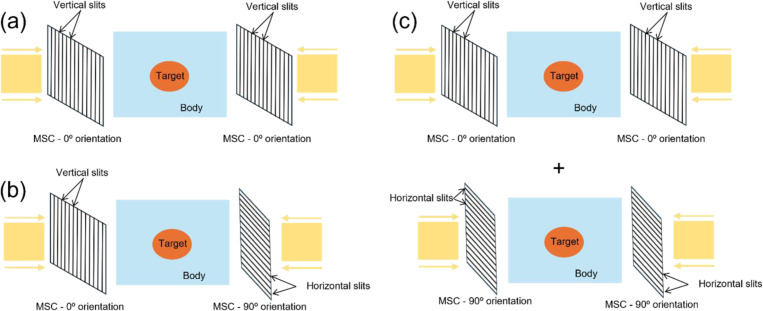
Three minibeam-pLATTICE models. (a) **M0** model with 0° MSC orientation for all beam angles, (b) **M1** model with alternating 0° and 90° MSC orientations, (c) **M2** model with both 0° and 90° MSC orientations at each angle.

**Table 1:** Comparison of conventional pLATTICE (CONV) and minibeam-pLATTICE (M0, M1, M2) for HN01 case. The prescribed peak and valley doses are 10 Gy and 2 Gy respectively.

Structure	Quantity	CONV	M0	M1	M2
CTV	Mean ${\text{D}}_{\text{peak}}$	15.14 Gy	11.69 Gy	11.48 Gy	11.64 Gy
	Mean ${\text{D}}_{\text{valley}}$	3.20 Gy	2.66 Gy	2.58 Gy	2.60 Gy
	PVDR	4.131	4.871	4.703 Gy	5.893

Body	${\text{D}}_{\text{mean}}$	0.197 Gy	0.140 Gy	0.139 Gy	0.142 Gy

Brain	${\text{D}}_{\text{mean}}$	0.518 Gy	0.565 Gy	0.562 Gy	0.531 Gy
	${\text{D}}_{\text{max}}$	3.931 Gy	7.203 Gy	8.109 Gy	6.524 Gy

Brainstem	${\text{D}}_{\text{mean}}$	0.028 Gy	0.035 Gy	0.032 Gy	0.032 Gy
	${\text{D}}_{\text{max}}$	16.574 Gy	6.543 Gy	7.047 Gy	5.805 Gy

**Table 2:** Comparison of conventional pLATTICE (CONV) and minibeam-pLATTICE (M0, M1, M2) for HN02 case. The prescribed peak and valley doses are 10.6 Gy and 2.12 Gy respectively.

Structure	Quantity	CONV	M0	M1	M2
CTV	Mean ${\text{D}}_{\text{peak}}$	12.31 Gy	11.72 Gy	11.91 Gy	11.70 Gy
	Mean ${\text{D}}_{\text{valley}}$	3.76 Gy	3.15 Gy	3.41 Gy	3.14 Gy
	PVDR	3.426	2.050	2.310	2.555

Body	${\text{D}}_{\text{mean}}$	0.036 Gy	0.032 Gy	0.033 Gy	0.032 Gy

Larynx	${\text{D}}_{\text{mean}}$	0.242 Gy	0.288 Gy	0.285 Gy	0.272 Gy
	${\text{D}}_{\text{max}}$	2.273 Gy	2.605 Gy	2.531 Gy	2.529 Gy

Oral	${\text{D}}_{\text{mean}}$	0.227 Gy	0.173 Gy	0.169 Gy	0.179 Gy
	${\text{D}}_{\text{max}}$	2.097 Gy	2.525 Gy	2.880 Gy	2.841 Gy
